# Weekly taxane–anthracycline combination regimen versus tri-weekly anthracycline-based regimen for the treatment of locally advanced breast cancer: a randomized controlled trial

**DOI:** 10.1186/s40880-017-0196-5

**Published:** 2017-03-07

**Authors:** Qiu-Wen Tan, Ting Luo, Hong Zheng, Ting-Lun Tian, Ping He, Jie Chen, He-Lin Zeng, Qing Lv

**Affiliations:** 1 Department of Breast Surgery, West China Hospital, Sichuan University, Chengdu, 610041 Sichuan P. R. China; 2 Department of Medical Oncology, West China Hospital, Sichuan University, Chengdu, 610041 Sichuan P. R. China

**Keywords:** Locally advanced breast cancer, Neoadjuvant, Randomized clinical trial, Taxanes, Anthracycline, Chemotherapy

## Abstract

**Background:**

Extensive studies have confirmed the efficacy of taxanes in combination with anthracycline-based chemotherapy on breast cancer. However, few studies have assessed the efficacy of weekly taxane–anthracycline regimens on locally advanced breast cancer. This study was to compare the efficacy and safety of a weekly taxane–anthracycline regimen with those of tri-weekly anthracycline-based regimen in patients with locally advanced breast cancer.

**Methods:**

Patients with locally advanced breast cancer were randomized to receive 4–6 cycles of neoadjuvant chemotherapy with tri-weekly 5-fluorouracil–epirubicin–cyclophosphamide (FEC) regimen or weekly paclitaxel–epirubicin (PE) regimen. The primary endpoint was the pathologic complete response (pCR) rate. Other endpoints included the clinical tumor response, breast-conserving surgery rate, and adverse events.

**Results:**

Between March 2010 and September 2013, 293 patients were randomized to the FEC (*n* = 151) and PE (*n* = 142) arms. The overall clinical response rate was significantly higher in the PE arm than in the FEC arm (76.06% vs. 59.95%, *P* = 0.001). Consistently, the post-chemotherapy pathologic T and N stages were significantly lower in the PE arm than in the FEC arm (*P* < 0.001). However, the pCR rate was similar in the two arms (10.61% vs. 12.31%, *P* = 0.665). Overall, 36 (27.27%) patients in the FEC arm and 6 (35.28%) in the PE arm were qualified for breast-conserving surgery. Most adverse events were comparable in both arms, with more severe neutropenia in the PE arm than in the FEC arm (11.97% vs. 5.96%, *P* = 0.031).

**Conclusions:**

In patients with locally advanced breast cancer, weekly PE was not superior to FEC in terms of pCR. However, weekly PE has a higher response rate and superior down-staging effects. On this account, the PE regimen may be considered an alternative option for locally advanced breast cancer. Long-term follow-up data are needed to confirm the efficacy of this regimen on locally advanced breast cancer.

*Trial registration* Chinese clinical trial registry, ChiCTR-TRC-10001043, September 21, 2014

## Background

Locally advanced breast cancer is a heterogeneous entity that includes advanced primary tumors, extensive nodal involvement, and inflammatory breast cancer [1, 2]. Despite the progress in understanding tumor biology and the development of targeted therapy, locally advanced breast cancer remains a major clinical challenge with an unfavorable prognosis [1]. Neoadjuvant chemotherapy (NACT) is a standard treatment of locally advanced breast cancer [3, 4]. Women who achieved a pathologic complete response (pCR) during NACT had prolonged survival compared with those who did not achieved pCR [5].

Anthracycline-based regimens are the most effective chemotherapy for breast cancer [6]. Anthracycline-based regimens, such as tri-weekly 5-fluorouracil–epirubicin–cyclophosphamide (FEC) regimen, is widely recommend by guidelines and used in clinical practice. The addition of taxanes to anthracycline-based regimens has been shown to enhance antitumor activity with increased pCR and breast-conserving surgery (BCS) rate as well as prolonged survival [7, 8]. In addition, the Eastern Cooperative Oncology Group (ECOG) 1199 trial demonstrated a significant disease-free survival benefit of weekly paclitaxel [9, 10]. Theoretically, the ideal chemotherapy regimen should be safe, effective, and simple. For these reasons, weekly paclitaxel–epirubicin (PE) regimen is attractive. In addition, considering the mild myelosuppressive effect of paclitaxel, weekly PE could be a convenient outpatient chemotherapy regimen.

Till now, extensive studies have evaluated the therapeutic value of taxanes in combination with anthracycline-based chemotherapy in breast cancer; however, limited data are available to evaluate its efficacy on locally advanced breast cancer, especially in Chinese women. In this prospective randomized controlled trial, we compared the safety and efficacy of a weekly PE regimen with those of the tri-weekly FEC regimen in Chinese women with locally advanced breast cancer.

## Patients and methods

### Participant enrollment

This prospective randomized controlled trial was approved by the Ethics Committee of West China Hospital of Sichuan University. Written informed consent was obtained from all participants. The study was registered with the Chinese Clinical Trial Register on September 21, 2014 (Registration number: ChiCTR-TRC-10001043).

Women aged 18–70 years old with locally advanced breast cancer confirmed by core needle biopsy were eligible for our study. Locally advanced breast cancer was classified as clinical stage IIB or III according to the American Joint Committee on Cancer staging system. Before randomization, baseline chest radiography, abdominal computed tomography (CT) or magnetic resonance imaging, and bone scintigraphy were performed to exclude distant metastases. Other eligibility criteria were an ECOG performance status of 0–1; normal cardiac function; no history or evidence of abnormal hematologic, renal, or hepatic function; and no history of other neoplasm (except non-melanoma skin cancer or curatively treated carcinoma in situ of the cervix).

Patients were excluded if they were pregnant; had received prior breast cancer surgery or systemic therapy; had uncontrolled concurrent illness such as serious viral, bacterial, or fungal infections, peptic ulcers or diabetes, or autoimmune diseases; had a history of severe hypersensitivity reactions to chemotherapeutic regimens; or had any other illness deemed by the physician to affect chemotherapy tolerability.

### Treatment

With simple randomization, the participants were randomly assigned to the PE arm or the FEC arm. In the PE arm, intravenous infusion of epirubicin 30–40 mg/m^2^ and paclitaxel 70–80 mg/m^2^ were administered concurrently on days 1, 8, and 15 of every 4-week cycle. In the FEC arm, intravenous infusion of 5-fluorouracil 500 mg/m^2^, epirubicin 100 mg/m^2^, and cyclophosphamide 500 mg/m^2^ were administered on day 1 of every 3-week cycle. Antimimetic drugs were administered prophylactically 30 min before the chemotherapeutic regimen was administered. During NACT, granulocyte colony-stimulating factor (G-CSF) support was required if the neutrocyte count dropped to <1.0 × 10^9^/L.

Surgery was undertaken within 1–2 weeks of NACT completion. According to the tumor characteristics and patient preference, women underwent BCS or mastectomy. Those considered eligible for BCS had a single tumor <3 cm in diameter, with the distance between the tumor edge and nipple being ≥3 cm, had no diffuse lesion or skin involvement, and were not contraindicated for radiotherapy. All patients who underwent BCS also underwent postoperative radiotherapy. All these patients underwent axillary lymph node dissection for nodal assessment.

All study visits were completed at the Breast Cancer Center of West China Hospital. At the beginning of each cycle, history taking, physical examination, and hematologic assessment were conducted to evaluate safety. The NACT schedule was delayed if the left ventricle ejection fraction (LVEF) decreased by 15% or if the patient showed symptoms of congestive heart failure, a severe hypersensitive reaction, or other adverse events during treatment. In the PE arm, if the patients had severe neutropenia (neutrophil count <1.0 × 10^9^/L), febrile neutropenia (grade 2 and above), or peripheral neuropathy (grade 2 and above), the dose of epirubicin and paclitaxel was reduced by 15%.

Participants were withdrawn if they had disease progression or developed severe adverse events (e.g., grade 3 or 4 non-hematologic toxicity), or at their request.

### Efficacy assessments

Physical examination and imaging data (ultrasonography for tumor response assessment and CT scan for metastasis monitoring) were carefully recorded for clinical assessments before treatment, every two cycles during NACT, and before surgery. The tumor response was assessed by experienced oncologists and was classified as clinical complete response (cCR), partial response (cPR), stable disease (cSD), or progressive disease (cPD). In particular, the clinical tumor response was defined as the achievement of cCR and cPR. Any controversy was solved by discussion with a third oncologist. Tumor responses were used to dictate management strategies. Those showing a cCR, defined as the disappearance of the breast tumor and enlarged nodes on clinical assessments, could undergo surgery. Those showing a cPR, defined as a reduction of ≥30% in the three largest perpendicular tumor diameters, could complete at least four NACT cycles and then undergo surgery. Those with cSD, defined as a tumor reduction of <30%, or those with cPD, defined as an increase of ≥20% in the target tumor diameter or the emergence of a new tumor, could switch NACT regimens or undergo surgery as desired.

Postoperative pathologic assessments were conducted by pathologists at the Pathology Department of West China Hospital. A pCR was defined as the complete disappearance of the invasive tumor in the breast and lymph nodes. Residual ductal carcinoma in situ (DCIS) alone was also classified as pCR. Before the assessments and data analysis, the two groups were renamed as group 1 or 2 without detailed information on the NACT provided. Therefore, the surgeons who assessed the suitability for BCS, the pathologists who assessed the postoperative specimens, and the statisticians who performed the analysis were all blinded.

### Safety assessments

All adverse events were recorded and graded according to the National Cancer Institute Common Toxicity Criteria (version 2.0). All women who underwent at least 1 cycle of chemotherapy were included in the safety analysis. Only grade 3–4 adverse events were analyzed.

### Statistical analyses

The sample size was estimated to detect a pCR rate difference of 25% for the PE arm and 10% for the FEC arm. The assumed dropout rate was 10%. A sample size of 218 participants was sufficient to provide an 80% power to detect a pCR improvement of 15% in each arm with a type I error rate of 0.05.

All data were analyzed based on the intent-to-treat principle at randomization. Descriptive data were used to analyze patient characteristics. Quantitative data were compared using an independent sample *t* test, and qualitative data were compared using the Chi square test. Ranked data were compared using a non-parametric test. Statistical tests were considered significant with a two-sided *P* value of <0.05. All data were analyzed using SPSS version 16.0 (SPSS Inc., Chicago, IL, USA).

## Results

### Patient characteristics

**Fig. 1:**
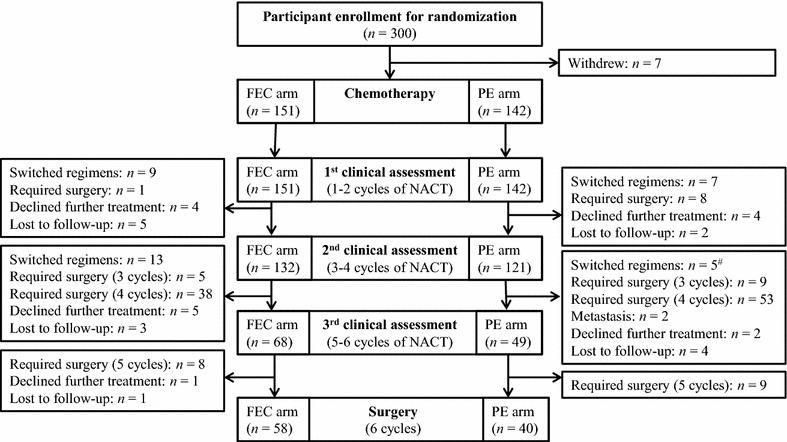
Study flow chart for comparison of weekly PE and tri-weekly FEC regimens in treating locally advanced breast cancer. *FEC* tri-weekly 5-fluorouracil–epirubicin–cyclophosphamide regimen, *PE* weekly paclitaxel–epirubicin regimen, *NACT* neoadjuvant chemotherapy. ^#^All participants who switched chemotherapy regimens had undergone surgery except one in the PE arm that was lost to follow-up

The results of randomization and treatment assignment are shown in Fig. 1. Between March 2010 and September 2013, 300 patients were enrolled, but 7 of them withdrew prior to treatment. Of the remaining 293 patients, 151 were assigned to the FEC arm, and 142 were assigned to the PE arm. The baseline patient characteristics of the two groups were evenly matched and are shown in Table 1. The median ages were 47 (range 27–69) years for the FEC arm and 47 (range 24–68) years for the PE arm. During chemotherapy, 16 patients (10 in the FEC arm and 6 in the PE arm) had declined further treatment; 15 (9 in the FEC arm and 6 in the PE arm) were lost to follow-up; and 34 (22 in the FEC arm and 12 in the PE arm) had switched chemotherapy regimens due to unsatisfactory outcomes or toxicities.

**Table 1:** Baseline characteristics of all enrolled patients with locally advanced breast cancer

Characteristic	FEC arm [cases (%)]	PE arm [cases (%)]	*P* value
Total	151	142	
Age (years)			0.430
≤35	5 (3.31)	8 (5.63)	
>35	146 (96.69)	134 (94.37)	
Menopausal status			0.263
Premenopausal	31 (20.53)	22 (15.49)	
Postmenopausal	120 (79.47)	120 (84.51)	
Clinical tumor stage			0.075
T2	106 (70.20)	86 (60.56)	
T3	43 (28.48)	52 (36.62)	
T4	2 (1.32)	4 (2.82)	
Clinical nodal status			0.497
Involved	130 (86.09)	126 (88.73)	
Not involved	21 (13.91)	16 (11.27)	
ER/PR status			0.533
Positive	96 (63.58)	89 (62.68)	
Negative	47 (31.13)	52 (36.62)	
Missing	8 (5.30)	1 (0.70)	
HER2 (IHC staining)			0.189
0/1+	64 (42.38)	66 (46.48)	
2+	31 (20.53)	26 (18.31)	
3+	45 (29.80)	47 (33.10)	
Missing	11 (7.28)	3 (2.11)	

*PE* weekly paclitaxel–epirubicin regimen, *FEC* tri-weekly 5-fluorouracil–epirubicin–cyclophosphamide regimen, *ER* estrogen receptor, *PR* progesterone receptor, *HER2* epidermal growth factor receptor-2, *IHC* immunohistochemistry

### Clinical response

**Fig. 2:**
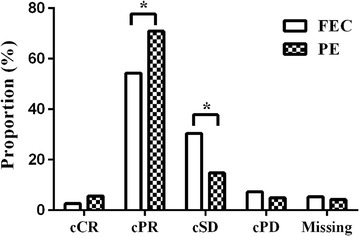
Final assessment of clinical responses of patients with locally advanced breast cancer to neoadjuvant chemotherapy (FEC regimen vs. PE regimen). *FEC* tri-weekly 5-fluorouracil–epirubicin–cyclophosphamide regimen, *PE* weekly paclitaxel–epirubicin regimen, *cCR* clinical complete response, *cPR* clinical partial response, *cSD* clinical stable disease, *cPD* clinical progressive disease. *P < 0.05

During chemotherapy, three assessments were performed to grade the clinical response to these two regimens (Table 2). The results of the last assessment for individual participants are shown in Fig. 2. The clinical response rates were significantly higher in the PE arm than in the FEC arm (76.55% vs. 56.95%, *P* = 0.001). Those in the PE arm achieved higher cPR and lower cSD rates than those in the FEC arm (cPR rate: 70.92% vs. 54.30%, *P* = 0.006; cSD rate: 14.79% vs. 30.46% *P* = 0.002). Two patients in the PE arm developed distant metastasis after 4 cycles of NACT.

**Table 2:** Clinical responses of patients with locally advanced breast cancer to neoadjuvant chemotherapy (FEC regimen vs. PE regimen) during each assessments

Group	First assessment [cases (%)]	Second assessment [cases (%)]	Third assessment [cases (%)]
FEC arm	151	132	68
cCR	0 (0.00)	1 (0.76)	3 (4.41)
cPR	70 (46.36)	76 (57.58)	51 (75.00)
cSD	67(44.37)	45 (34.09)	13 (19.12)
cPD	6 (3.97)	7 (5.30)	1 (1.47)
Missing	8 (5.30)	3 (2.27)	0 (1.47)
PE arm	142	121	49
cCR	1 (0.70)	6 (4.96)	5 (10.20)
cPR	97 (68.31)	92 (76.03)	41 (83.67)
cSD	35 (24.65)	20 (16.53)	2 (4.08)
cPD	3 (2.11)	3 (2.48)	1 (2.04)
Missing	6 (4.23)	0 (0.00)	0 (0.00)

*FEC* tri-weekly 5-fluorouracil–epirubicin–cyclophosphamide regimen, *PE* weekly paclitaxel–epirubicin regimen, *cCR* clinical complete response, *cPR* clinical partial response, *cSD* clinical stable disease, *cPD* clinical progressive disease

For each participant, the tumor was restaged at the last assessment. As shown in Table 3, both regimens exhibited excellent down-staging effects (both *P* < 0.001), with the PE regimen exhibiting a superior down-staging effect compared with the FEC regimen (*P* = 0.026).

**Table 3:** Clinical down-staging effects of the FEC and PE regimens on locally advanced breast cancer

T stage	FEC arm [cases (%)]		*P* value	PE arm [cases (%)]		*P* value
	Baseline	After NACT		Baseline	After NACT	
Total	151	151	<0.001	142	142	<0.001
0	0 (0.00)	5 (3.31)		0 (0.00)	12 (8.45)	
1	0 (0.00)	46 (30.46)		0 (0.00)	62 (43.66)	
2	106 (70.20)	77 (50.99)		86 (60.65)	55 (38.73)	
3	43 (28.48)	9 (6.62)		52 (36.62)	5 (3.52)	
4	2 (1.32)	5 (2.65)		4 (2.82)	2 (1.41)	
Missing	0 (0.00)	9^a^ (5.96)		0 (0.00)	6^b^ (4.23)	

*FEC* tri-weekly 5-fluorouracil–epirubicin–cyclophosphamide regimen, *PE* weekly paclitaxel–epirubicin regimen

^a^Of the 9 patients, 2 had migrated to other cities and were not restaged, 5 declined further treatment and were not restaged, and 2 were lost to follow-up

^b^Of the 6 patients, 5 declined further treatment and were not restaged, and 1 was lost to follow-up

### Surgery and pathologic response

Among the 293 patients, 262 underwent surgery (132 in the FEC arm and 130 in the PE arm). Among them, 219 patients (114 in the FEC arm and 105 in the PE arm) had completed at least 4 cycles of NACT; 33 (22 in the FEC arm and 11 in the PE arm) had switched chemotherapy regimens.

Surgery information is shown in Table 4. No significant difference was observed in the BCS rate. Theoretically, 36 (27.27%) patients in the FEC arm and 6 (35.38%) in the PE arm were candidates for BCS (*P* = 0.157). However, only 3 (2.27%) in the FEC arm and 4 (3.08%) in the PE arm underwent BCS (*P* = 0.721).

**Table 4:** Surgical breast and lymph node management for the FEC and PE arms

Surgery type	FEC arm [cases (%)]	PE arm [cases (%)]	*P* value
Total	132	130	
The breast			0.456
Breast-conserving surgery	3 (2.27)	4 (3.08)	
Mastectomy	75 (56.82)	66 (50.77)	
Modified radical mastectomy	54 (40.91)	60 (46.15)	
Lymph nodes			0.633
No surgery^a^	0 (0.00)	1 (0.77)	
SLNB^a^	1 (0.76)	2 (1.54)	
Level I–II node dissection	28 (21.21)	21 (16.15)	
Level I–III node dissection	86 (65.15)	89 (68.46)	
Level I–III + supraclavicular node dissection	17 (12.88)	17 (13.08)	

*FEC* tri-weekly 5-fluorouracil–epirubicin–cyclophosphamide regimen, *PE* weekly paclitaxel–epirubicin regimen, *SLNB* sentinel lymph node biopsy

^a^Required by participants

For pathologic response assessments, all the 262 patients were included in the intention-to-treat analysis. Postoperative pathologic staging indicated that the weekly PE regimen significantly alleviated tumor burden in patients with locally advanced breast cancer as compared with the tri-weekly FEC regimen (*P* = 0.001) (Table 5). However, no significant differences between the FEC and PE arms were observed in the overall pCR (pCR in both the breast and lymph nodes) rates (10.61% vs. 12.31%, *P* = 0.665), the breast pCR rates (13.64% vs. 16.92%, *P* = 0.460), or regional lymph node pCR rates (34.85% vs. 39.23%, *P* = 0.463).

**Table 5:** Postoperative pathologic staging in patients who underwent surgery

Pathologic stage	FEC arm [cases (%)]	PE arm [cases (%)]	*P* value
Total	132	130	
pT stage			0.001
0	11 (8.33)	15 (11.53)	
DCIS	7 (5.30)	7 (5.38)	
1	40 (30.30)	62 (47.69)	
2	57 (43.18)	41 (31.54)	
3	2 (1.52)	2 (1.54)	
4	15 (11.36)	3 (2.31)	
pN stage			0.001
0	46 (34.85)	51 (39.23)	
1	39 (29.55)	39 (30.00)	
2	25 (18.94)	18 (13.85)	
3	22 (16.67)	22 (16.92)	

*FEC* tri-weekly 5-fluorouracil–epirubicin–cyclophosphamide regimen, *PE* weekly paclitaxel–epirubicin regimen, *DCIS* ductal carcinoma in situ

### Safety

For the safety analysis, we evaluated only grade 3–4 adverse events, which are listed in Table 6. Both the FEC and PE regimens were well tolerated, and all adverse events were manageable. The frequency of neutropenia was higher in the PE arm than in the FEC arm (*P* = 0.031). Four patients (2 in each arm) had grade 4 neutropenia and were treated by repeated G-CSF administration. Three patients (2 in the FEC arm and 1 in the PE arms) had liver damage and required breast cancer surgery after 3 or 4 cycles of NACT. One patient had grade 4 bone marrow suppression after 1 cycle of PE and thus switched to the FEC regimen. There were no cardiac events or treatment-related deaths during the study period. Other adverse events were mild and slightly affected patient quality of life during NACT.

**Table 6:** Frequency of grade 3–4 adverse events in the FEC and PE arms

Adverse event	FEC arm [cases (%)]	PE arm [cases (%)]	*P* value
Hematologic			
Neutropenia	9 (5.96)	17 (11.97)	0.031
Anemia	6 (4.00)	10 (7.04)	0.248
Thrombocytopenia	5 (3.31)	7 (4.93)	0.485
Non-hematologic			
Nausea	4 (2.64)	4 (2.82)	0.458
Vomiting	2 (1.32)	3 (2.11)	0.603
Diarrhea	2 (1.32)	4 (2.82)	0.951
Constipation	3 (1.99)	4 (2.82)	0.267
Hair loss	24 (15.79)	28 (19.72)	0.583
Dermatitis	8 (5.30)	11 (7.75)	0.395
Febrile	1 (0.66)	1 (0.70)	0.965
Fatigue	7 (4.64)	10 (7.04)	0.379
Hand-foot syndrome	6 (3.97)	5 (3.52)	0.913
Allergy	0 (0.00)	1 (0.70)	0.143

*FEC* tri-weekly 5-fluorouracil–epirubicin–cyclophosphamide regimen, *PE* weekly paclitaxel–epirubicin regimen

## Discussion

Our results showed that the weekly PE regimen was not superior to the tri-weekly FEC regimen in treating locally advanced breast cancer in terms of pCR. However, in the NACT setting, the weekly PE regimen showed significant value in the clinical tumor response and down-staging effect.

In China, delayed detection and a lack of awareness of breast cancer have led to a high prevalence of locally advanced breast cancer at the initial diagnosis [11, 12]. Locally advanced breast cancer with large tumor lesions and more node involvement is related with a lower pCR rate as compared with early stage or operable breast cancer [13, 14]. In the present study, the overall pCR rate (12.31%) in the PE arm was comparable to those reported previously for taxane–anthracycline-based regimens (13.3%–18%) [15, 16]. Specifically, the results were similar to those of the ABCSG-14 trial, which administered 6 cycles of epirubicin 75 mg/m^2^ plus docetaxel 75 mg/m^2^, resulting in overall and breast pCR rates of 15.9% and 18.6%, respectively [17]. However, the ABCSG-14 trial included patients with diseases at any tumor stage, whereas the present study focused on those with locally advanced breast cancer who may have a poorer prognosis. Additionally, the predominant population in the present study was positive for hormone receptor, which was a predictive factor for a low response to NACT [18, 19].

In the present study, the pCR rate was higher in the PE arm than in the FEC arm, although the difference was not significant. However, we must note that more patients in the FEC arm switched regimens during the study than those in the PE arm (22 vs. 12). When performing the intention-to-treat analysis, the high regimen switch rate in the FEC arm was a confounder and an indirect indicator of poor disease control.

Although our findings do not support that the weekly PE regimen is superior than the tri-weekly FEC regimen in terms of the pCR rate, the PE regimen elicited a superior tumor response and down-staging effect in terms of the clinical and pathologic evaluations. Our results suggest that the weekly PE regimen can improve disease control and reduce the extent of surgical resection.

The increased chance of BCS is an important benefit of NACT. However, BCS might be difficult for women with locally advanced breast cancer, especially Chinese women. In the present study, the theoretical BCS rates were 27.27% in the FEC arm and 35.38% in the PE arm, which were lower than that reported by Amat et al. (overall BCS rate of 72.37%) [20]. These disparities could be caused by the inclusion of women with breast cancer at different stages in their study. The relatively small breast volume in Chinese women may also contribute to this phenomenon. In the present study, many patients who were qualified for BCS declined the surgery. Different attitudes towards breast cancer in eastern and western countries may explain the lower acceptance rate of BCS among Chinese women. In addition, the economic burden of postoperative radiotherapy and long-term follow-up might contribute to the preference of mastectomy over BCS [21–23].

In the present study, the adverse events were comparable in both arms, with more severe neutropenia in the PE arm, which could be successfully treated using G-CSF in our study and other studies [24, 25].

There are some limitations in the present study. Most epidermal growth factor receptor-2 (HER2)-positive patients in our study could not afford HER2-targeting therapy and refused further HER2 status testing. With this lack of data, we could not carry out subgroup analyses to identify any subpopulation that was more likely to benefit from the weekly PE regimen. Because there were no significant differences in terms of the pCR rate at the time of surgery, long-term follow-up data will be reported to further assess the efficacy of the weekly PE regimen on locally advanced breast cancer.

## Conclusions

This prospective, randomized study suggests that the weekly PE regimen is not superior to the tri-weekly FEC regimen in treating locally advanced breast cancer in terms of pCR. However, the weekly PE regimen is well tolerated and has a superior clinical tumor response in Chinese women with locally advanced breast cancer. Long-term outcomes are needed to confirm the efficacy of weekly taxanes in combination with an anthracycline-based regimen.
